# Teaching Potential of Multidisciplinary Tumor Board Meetings for Orthopedic Residents: Insights From a French Sarcoma Reference Center

**DOI:** 10.7759/cureus.39783

**Published:** 2023-05-31

**Authors:** Louis-Romée Le Nail, Ramy Samargandi

**Affiliations:** 1 Department of Orthopedics Surgery and Traumatology, Centre Hospitalier Régional Universitaire (CHRU) de Tours, Tours, FRA; 2 Groupe Innovation et Ciblage Cellulaire, Université de Tours, Tours, FRA; 3 Department of Orthopedic Surgery, Faculty of Medicine, University of Jeddah, Jeddah, SAU

**Keywords:** sarcoma, medical education, learning opportunities, musculoskeletal oncology, reference center, orthopedic residents, teaching, multidisciplinary tumor board meeting

## Abstract

Multidisciplinary tumor board meetings (MTBMs) have been established in oncology to improve patient care. While their benefits for patients have been well-documented, there are no previous studies on the teaching opportunities they provide for residents and medical students. This study aimed to examine the teaching provided to residents during MTBM and identify areas for improvement. The study hypothesized that although the primary objective of MTBM is not teaching, they could still contribute to residents' learning. The study focused on the tumors board meeting for bone metastasis and musculoskeletal tumors/sarcoma in a French reference center for musculoskeletal oncology. The composition of the MTBM included specialists from various disciplines, and it convened on a weekly basis to discuss cases. The orthopedic oncology residents (OORs) actively participated in the MTBM as part of their training. A questionnaire was distributed to OORs who had served between 2014 and 2021, and their responses were analyzed. The results showed that the OOR perceived MTBM as interesting, beneficial for their training, and challenging. While opinions were divided on whether MTBM was a suitable place for education, the majority believed it improved their knowledge of different specialties and provided valuable elements applicable to daily practice. They also felt it facilitated future access to MTBs if needed. OORs recommended the rotation as an orthopedic oncology resident to younger residents. Recommendations for improvement included incorporating more dedicated teaching time, active involvement of residents in the learning process, availability of learning materials, and post-MTBM debriefing sessions. The study highlighted the importance of case presentations, public speaking skills, interdisciplinary collaboration, and clinical reasoning observed during MTBM. In conclusion, while not primarily designed for teaching, MTBM offers valuable learning opportunities for residents. Enhancing the teaching experience through dedicated debriefing sessions, resident involvement, and improved support can further optimize their educational impact. Future evaluations are needed to assess the effectiveness of these improvements. This study provides insights into the teaching potential within MTBM and can guide educational initiatives in the field of oncology.

## Introduction

Multidisciplinary tumor boards (MTB) have been established in oncology in France since 2003 [1]. Their benefits for patients, particularly in terms of overall survival, have been demonstrated [2]. In 2017, the French Ministry of Health defined the MTB in a Continuing Professional Development document as follows: "Multidisciplinary tumor board meetings (MTBM) facilitate collaboration among healthcare professionals from diverse specialties, pooling their expertise to make informed decisions that provide patients with the best possible care based on the current state of knowledge" [3]. This document outlines the definition, composition, and essential operational elements of the MTB. However, it does not mention any educational mission or teaching opportunities within these meetings.

The medical literature contains numerous comprehensive studies and recommendations focusing on the operational aspects of MTBM and the benefits they offer to patients whose cases are discussed [4-7]. However, the literature has not yet addressed the potential teaching opportunities that MTBM could provide for residents and medical students. The highly specialized nature of MTBM offers a unique chance for educational enrichment, allowing residents and medical students to enhance their understanding regarding this specific domain.

To the best of our knowledge, there are no published studies that have assessed the various teaching opportunities within MTBM. Thus, the aim of this study was to examine the teaching provided to residents during MTBM and identify areas for potential improvement. We hypothesized that although the primary objective of MTBM is not teaching, they could still contribute to residents' learning. The findings of this study could guide the development and implementation of educational initiatives that maximize the teaching potential within MTB, ultimately enhancing the education of residents and medical students in the field of oncology.

## Materials and methods

Multidisciplinary tumor board meetings (MTBMs) in our institution

Sarcomas are rare malignant tumors that originate from mesenchymal cells. In France, the annual incidence is approximately 4,000 new cases [8]. These tumors can develop in either the bone or soft tissues, with the limbs being the primary location [9]. The main treatment approach, when feasible, involves wide resection, which is often complex. This is typically accompanied by neo-adjuvant or adjuvant radiotherapy and sometimes chemotherapy [10,11].

According to the recommendations of the French National Cancer Institute (INCa), sarcoma management should be conducted in a designated reference center. The MTBM for bone metastasis and musculoskeletal tumors/sarcoma at the University Hospital of Tours has been in existence for about 30 years. It holds the INCa label and is a member of the French Sarcoma Network (NETSARC), previously known as the French Sarcoma Group/Bone Tumor Study Group. At the University Hospital of Tours, the MTBM is coordinated by orthopedic surgeons specializing in musculoskeletal oncology. The MTBM composition includes musculoskeletal oncology surgeons, a radiologist specializing in musculoskeletal imaging, a specialized sarcoma pathologist, medical oncologists, radiation oncologists, rheumatologists, plastic surgeons, and a medical secretary.

The MTBM is conducted weekly, discussing approximately 2,000 to 3,000 cases each year, encompassing musculoskeletal sarcomas and bone metastases. Prior to each case discussion, thorough preparation is undertaken, ensuring the completeness of administrative data, clinical information, and patient imaging. These essential details are then put into specialized oncology software within the Center of Val de Loire region, known as the shared Cancerology File. Following the MTBM, a comprehensive letter is composed and distributed to various correspondents, including the referring physician, the patient's primary care physician, and the physician responsible for the patient's ongoing care. This letter serves the purpose of conveying the management decision reached during the MTBM.

Orthopedic oncology residents

MTBM occupies a significant part of the residents’ activities, encompassing various responsibilities such as consultations, operating room procedures, surgical ward duties, and follow-up care. Specifically, orthopedic residents at our orthopedic department are assigned to MTBM on a rotational basis, typically lasting three months. In 2014, the position of orthopedic oncology resident (OOR) was established to further enhance the team. The OOR is entrusted with several key responsibilities, including active involvement in file preparation and presentation during MTBM sessions, drafting concise summary letters, participating in complex and demanding surgical interventions, performing less complex procedures under supervision, and assisting in the overall management of patient care within the department. 

**Figure 1:**
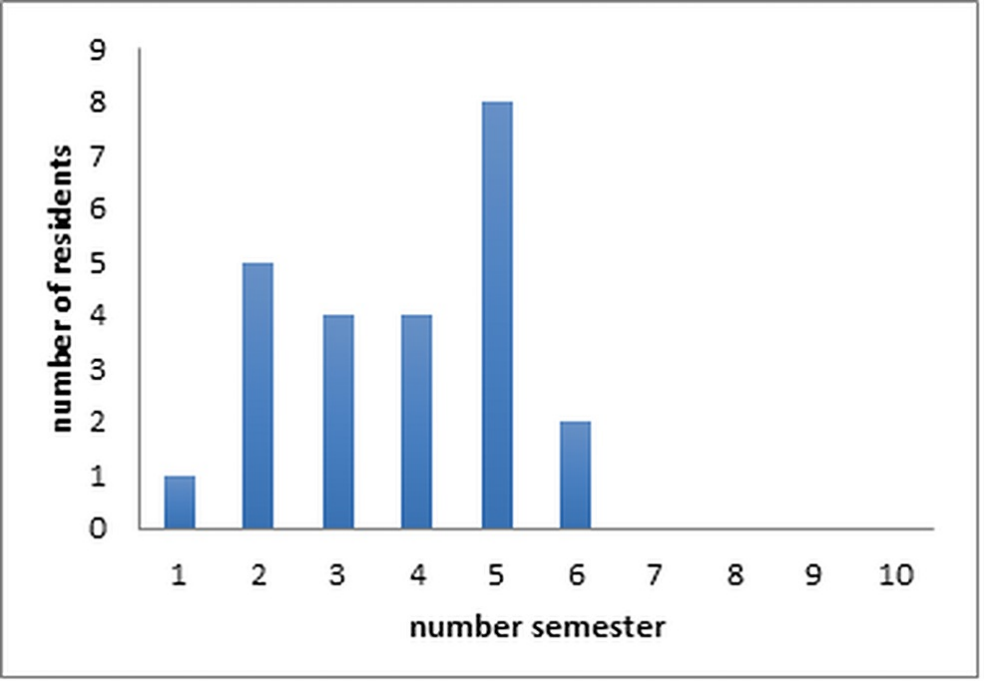
Semester numbers during which the 24 orthopedic residents, who responded, fulfilled the role of OOR. OOR: orthopedic oncology resident

This survey-based study investigates the experiences of 27 residents who served as Orthopedic Oncology Residents (OORs) between 2014 and 2021. Informed consent was obtained from all participants prior to completing and submitting the survey. The distribution of OORs based on their semester in the residency program is illustrated in Figure 1.

Survey

To ensure the face validity of the questionnaire, a panel of experts consisting of three individuals with extensive knowledge and experience in medical education and oncology was assembled. The experts were given access to the questionnaire and were asked to review its content, structure, and language to ensure that the items were clear, relevant, and comprehensible to the target population. Their valuable feedback and suggestions were incorporated into the final version of the questionnaire, thus enhancing its face validity.

The questionnaire (appendix 1) was then distributed to all orthopedic residents who had held the position of OOR. The questionnaire was sent via email, containing an internet link that directed the recipients to an online form questionnaire (Google docs; Google Inc., Mountain View, California, USA). Some residents had already completed their orthopedic residency and left our center during this time period. The survey focused solely on various aspects of the MTB and did not cover other related elements such as the orthopedic oncology outpatient department, surgeries, rounds, or weekly teaching. The questionnaire consisted of general and specific questions, and respondents provided their opinions using a Likert scale. All data collected were anonymized, and the results were compiled in an Excel spreadsheet for analysis (Microsoft Corporation, Richmond, Virginia, USA). Statistical analysis was conducted using median and range for continuous variables and number and percentage (%) for categorical variables. Surveys with incomplete data were excluded from the analysis. Out of the 27 orthopedic residents included in the study, 24 (89%) completed the questionnaire. The median semester of the residents was the 4th semester, with a range spanning from the 1st to the 6th semester.

## Results

Perception of MTBM by orthopedic oncology residents

**Figure 2:**
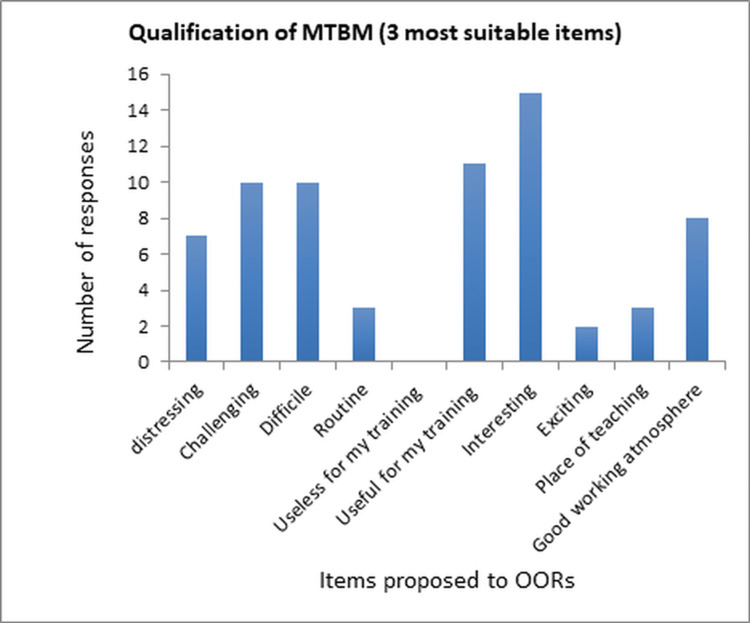
Perception of MTBM by OORs OORs: orthopedic oncology residents, MTBM: multidisciplinary tumor board meetings

The OORs were requested to select three items out of 11 options that most accurately depicted their perception of the MTBM (Figure 2). Among the 24 respondents, two OORs chose only one or two items instead of three. The MTBM was primarily viewed as interesting, beneficial for their training, and equally demanding and challenging.

**Figure 3:**
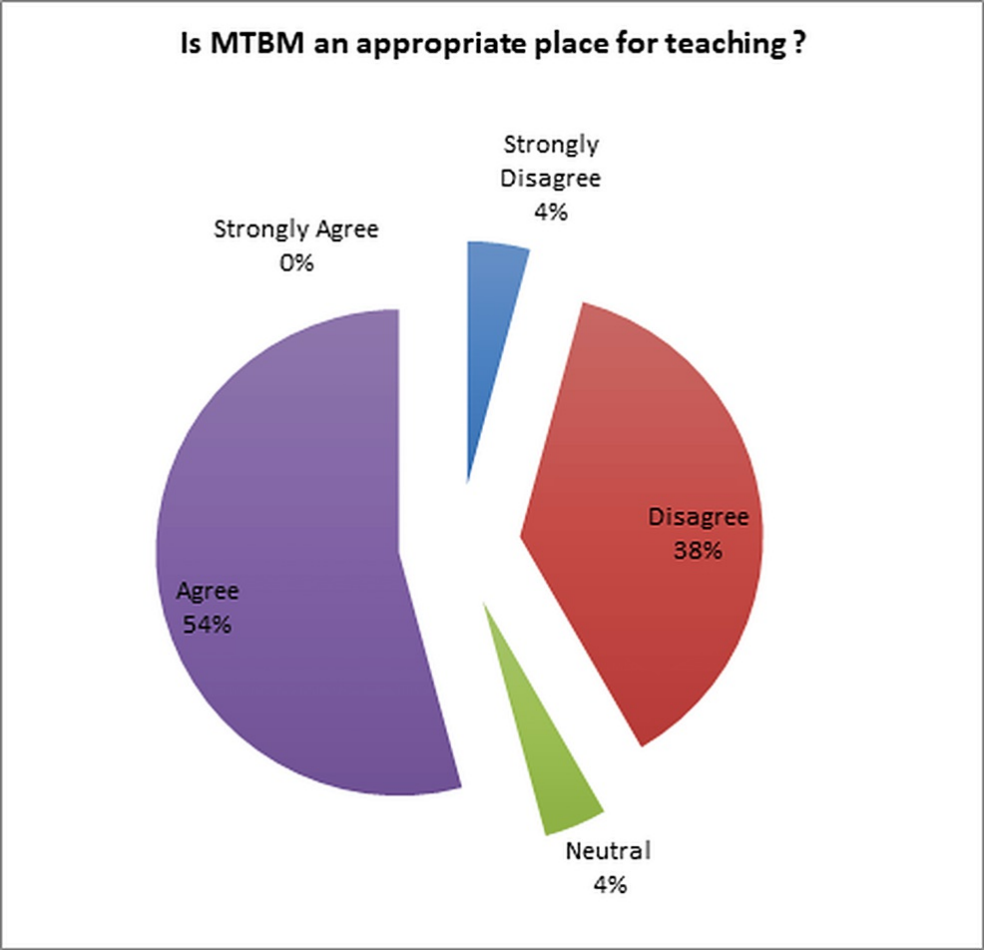
Evaluation of the perception of MTBM as a suitable place for education MTBM: multidisciplinary tumor board meetings

In response to the question, "Is MTBM an appropriate venue for teaching?" Opinions were divided among the participants. Approximately 54% expressed a favorable view (Figure 3), while 42% held an unfavorable opinion, and 4% had no specific opinion.

Acquired knowledge

**Figure 4:**
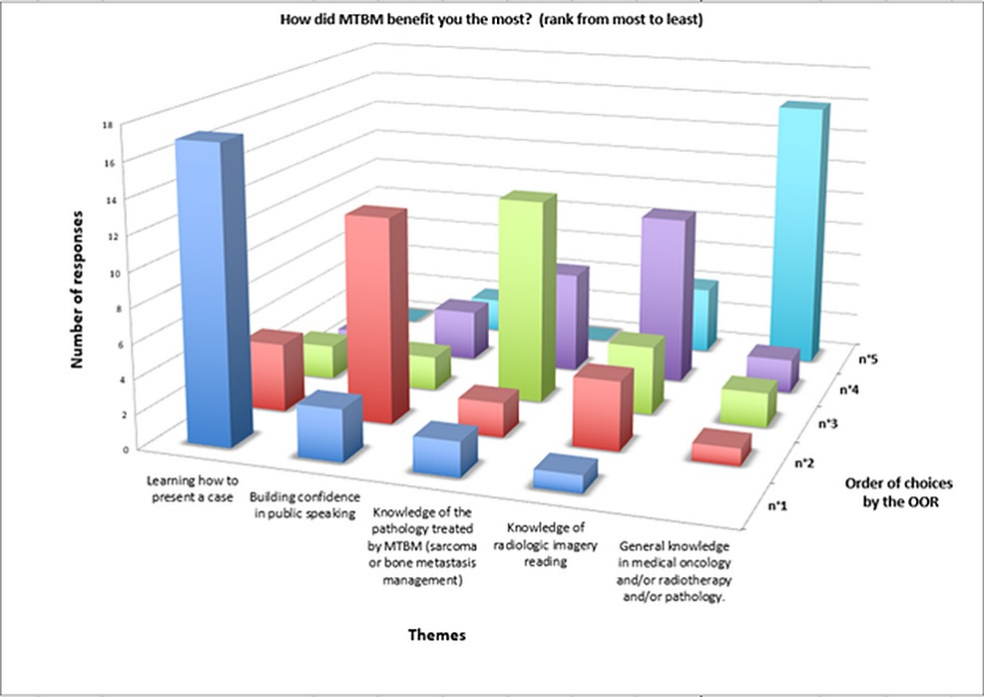
Ranking of learned themes OOR: orthopedic oncology resident, MTBM: multidisciplinary tumor board meetings

The OORs were provided with five themes and asked to prioritize them in terms of their perceived benefits (Figure 4). However, in two of the returned questionnaires, only one or two items were completed instead of the requested five. Among the completed responses, the highest priority was given to "learning how to present a case," selected by 71% (17/24) of the respondents. The second highest priority was "learning to speak in public," chosen by 52% (12/23) of the participants. The third priority was "knowledge of the different pathologies discussed in MTBM (sarcoma or bone metastases)," with 55% (12/22) selecting this option. "Knowledge in imaging reading and interpretation" followed closely with a majority of 45% (10/22). The final priority was "general knowledge in medical oncology and/or radiotherapy and/or pathology and/or rheumatology," which was chosen by 73% (16/22) of the respondents.

**Figure 5:**
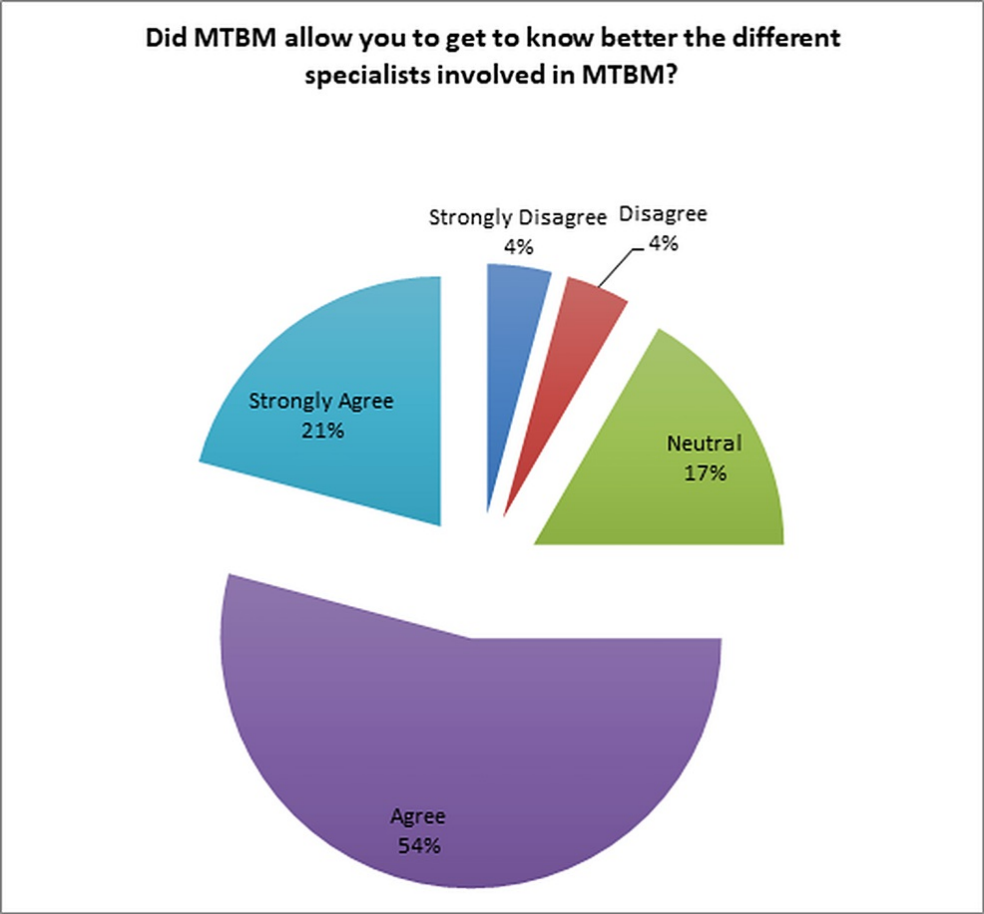
Knowledge of different specialties involved in MTBM MTBM: multidisciplinary tumor board meetings

When asked about the impact of MTBM on their knowledge of the various specialties involved, 75% (18/24) of the respondents responded positively (Figure 5).

**Figure 6:**
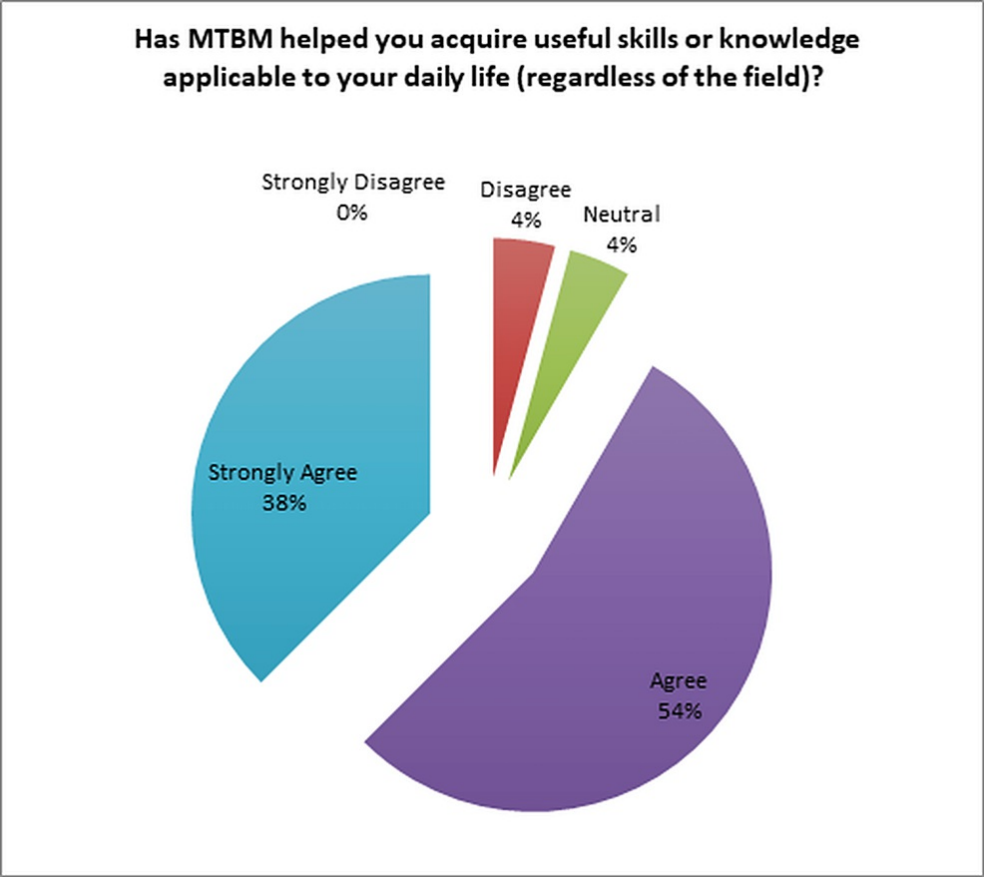
Acquisition of practical knowledge for everyday life MTBM: multidisciplinary tumor board meetings

**Figure 7:**
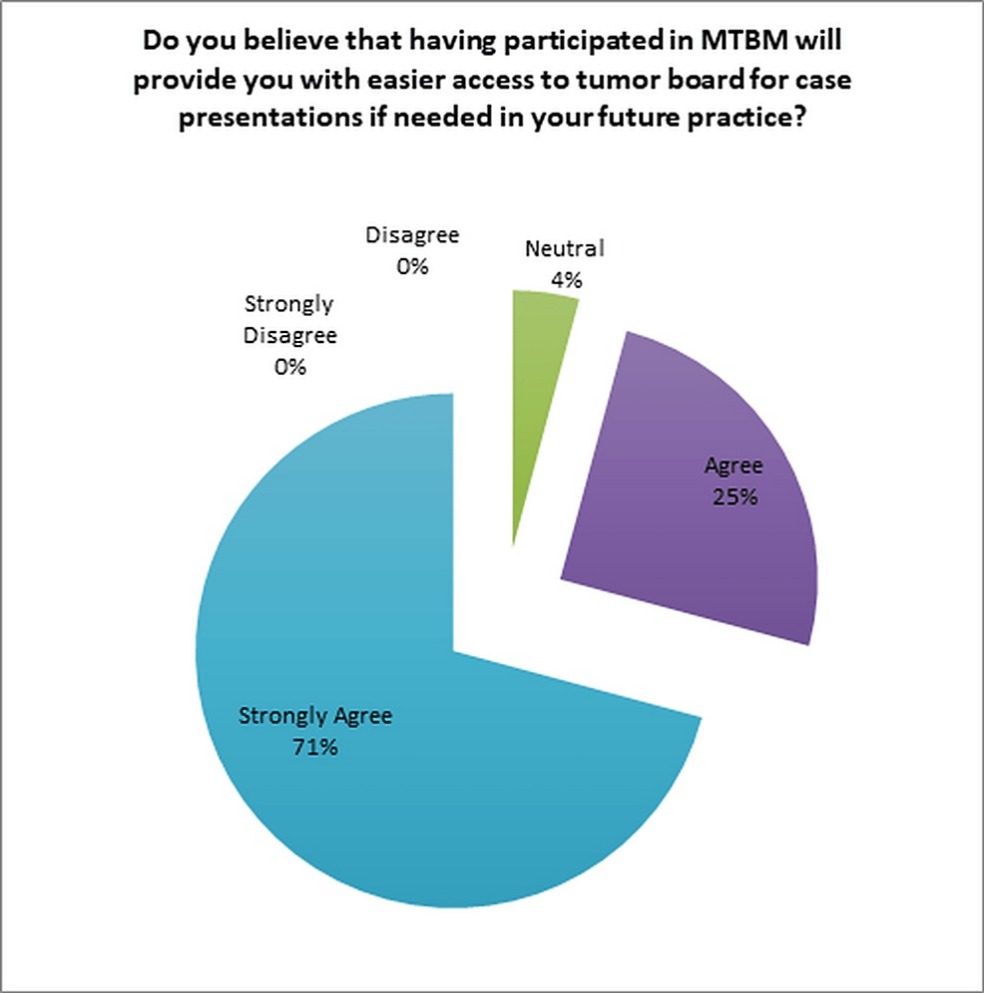
Evaluation of facilitation for future access to tumor board, if needed, for the residents. MTBM: multidisciplinary tumor board meetings

Moreover, a significant majority of the OORs, 92% (22/24), acknowledged that MTBM had provided them with valuable elements applicable to their daily practice, irrespective of their respective fields (Figure 6). Additionally, an overwhelming majority of 96% (23/24) of the OORs expressed that their participation in MTBM would make it easier for them to seek MTBM in the future, should the need arise (Figure 7).

Recommendations

**Figure 8:**
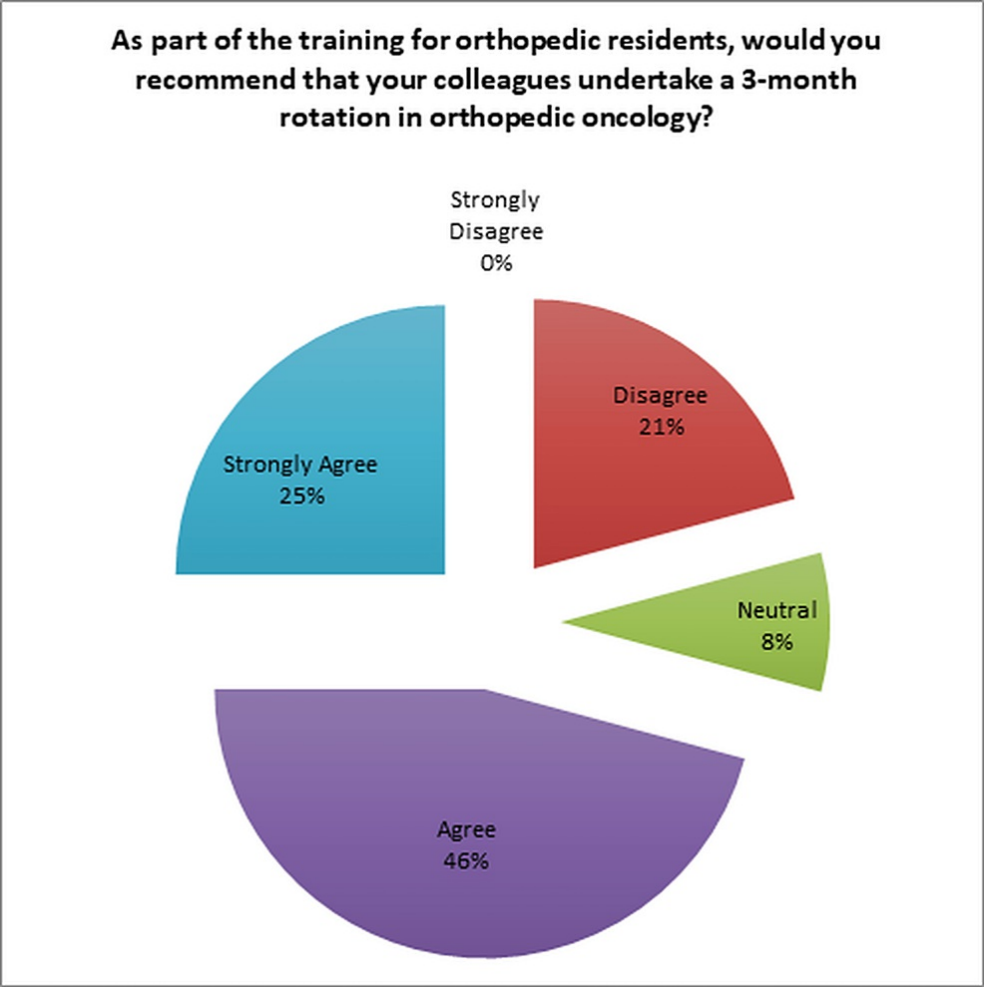
Recommendation to other orthopedic residents’ colleagues MTBM: multidisciplinary tumor board meetings

Regarding the question "As part of the training for orthopedic residents, would you recommend that your colleagues undertake a three-month rotation in orthopedic oncology?" as shown in Figure 8, the majority of respondents, 71% (17/24), responded positively and indicated that they would indeed recommend it.

Ways of improvement proposed by orthopedic oncology residents (OORs)

**Figure 9:**
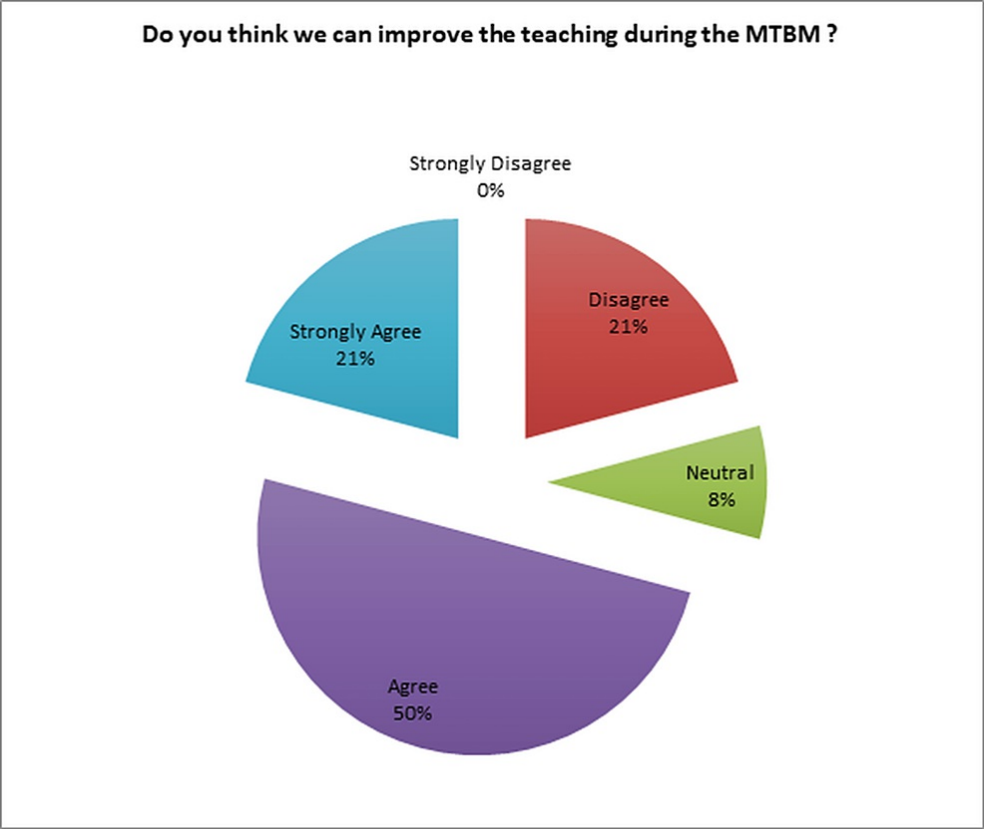
Opportunities for improvement in teaching during the MTBM MTBM: multidisciplinary tumor board meetings

Among the OORs, 71% (17/24) expressed their belief that there is room for improvement in teaching during MTBM, as shown in Figure 9.

Among the OORs, 21% (5/24) either did not have a proposed solution to improve teaching during MTBM or did not perceive a need for a solution. In contrast, 8% (2/24) of the OORs requested the availability of learning materials, which had not been implemented during their time as OORs, indicating a lack of specific learning resources for the OORs. Furthermore, 46% (11/24) of the OORs suggested that residents could actively participate in the learning process by being asked questions as part of formative assessment or engaging in quick case discussions where they explain their inquiries during the presentation of cases, all of which would take place during MTBM. An additional 4% (1/24) of the OORs proposed incorporating more dedicated teaching time during MTBM. Furthermore, 8% (2/24) of the OORs recommended post-MTBM debriefing sessions, while another 8% (2/24) suggested that the presence of all residents during MTBM would be beneficial.

## Discussion

Although MTBM was not specifically designed for teaching purposes, our study has uncovered several valuable insights. While there is currently no existing evidence in the medical literature on this topic, our findings from a single MTBM shed light on the learning opportunities it provides.

Firstly, OORs gained proficiency in delivering clear case presentations and improved their public speaking skills by presenting approximately 600 cases during their three-month rotation. They also learned how to write summary letters for the cases, including the transcription of the MTB's decision proposal, which were consistently reviewed by the musculoskeletal oncology surgeon responsible for MTBM.

Secondly, MTBM allowed orthopedic residents to develop a better understanding of the work environment in which they trained. It facilitated the establishment of connections and the building of relationships, emphasizing the value of teamwork and fostering mutual respect with physicians from other specialties. These intangible learning experiences were independent of the specific MTB theme. Additionally, OORs acquired general knowledge in other disciplines such as radiology, oncology, rheumatology, pathology, and radiotherapy during MTBM. This knowledge is essential for orthopedic residents, particularly in areas such as radiographic interpretation, rheumatology, basic oncology, and pathology. MTBM also demonstrated the value of open discussions among specialists, providing each participant with an opportunity to express their opinions in a supportive and collaborative atmosphere. It allowed residents to observe the clinical reasoning process employed by the multidisciplinary team in analyzing each case.

Finally, participating in MTBM enabled OORs to recognize situations where they should refer their future patients to a reference center in cases of suspected sarcomas, ensuring the provision of appropriate care. They became aware that inadequate care and treatment in non-reference centers can negatively impact patients' life expectancy [2,5,6,12]. As a result, 96% of OORs agreed that their participation in MTBM would facilitate the process of patient referral in their future practice. Despite some OORs finding certain aspects of MTBM challenging and exhausting, 71% of them would recommend to other residents to take on this position for three months to enhance their knowledge in orthopedic oncology and improve their noncognitive skills, including communication, public speaking, collaboration, teamwork, accountability, and respect.

In our opinion, there are numerous advantages to the OORs rotation, including opportunities for medical research, mastery of biopsy procedures and basic interventions, and assistance in the outpatient department. OORs have priority in participating in all musculoskeletal oncology surgical interventions during their three-month rotation period and receive support for performing basic surgical procedures. This study has allowed us to reflect on how we can enhance the teaching experience for residents during and throughout the MTB. The MTB requires significant and prolonged concentration from participating specialists, often lasting 3 to 4 hours, as the decisions made during the meeting have important consequences for the patients. Therefore, extending the MTBM to include dedicated teaching time for a small number of residents, potentially at the expense of patient care, presents a challenge.

After considering the suggestions provided by the OORs to enhance the teaching experience during the three-month rotation, we have developed the following plans for implementation. Firstly, we will allocate a dedicated debriefing session of approximately 10 minutes after the main MTBM, allowing OORs to ask any questions they may have. In the absence of specific questions, the teaching team will pose questions to facilitate learning. Secondly, since 2014, we have divided the MTBM into two parts due to the increased number of patients discussed. The second part focuses on analyzing patient files at the diagnostic stage and involves radiologists and orthopedic surgeons. During this session, OORs will be questioned on at least two patient cases, covering topics such as imaging interpretation, diagnostic approaches, differential diagnosis, and treatment plans. At the end of each meeting, learning materials and references will be provided for further reading, and any points requiring clarification will be discussed upon the residents' request. Thirdly, we aim to enhance the existing support provided to OORs by providing detailed information on logistic issues related to patient files and outlining the principles of patient care before they assume their role. Lastly, we plan to conduct a re-evaluation within two years to assess the effectiveness of these implemented improvements.

Despite certain limitations, it remains crucial to recognize the constraints inherent in our study. The evaluation was conducted retrospectively, encompassing variations in survey response timeframes and the duration of residents' rotations. The survey itself had its own set of limitations, including an overreliance on closed-ended questions and solely undergoing face validation through the transcription of questions and answers. Additionally, no objective assessment was conducted. Nevertheless, these limitations can be somewhat overlooked given that the study's primary focus was on residents' perceptions of learning opportunities during the MTBM, a topic that has not been previously explored, and the majority of responses were positive in nature.

The evaluation was performed through a single multidisciplinary meeting within a specific subspecialty. For example, the orthopedic surgery department of the University Hospital of Tours serves as a reference center for osteoarticular infections in the Great West of France (CRIOGO), holding weekly multidisciplinary meetings for osteoarticular infection cases involving radiologists, infectious disease consultants, microbiologists, and orthopedic surgeons. These multidisciplinary meetings also provide teaching opportunities for orthopedic residents in terms of diagnostic management approaches for osteoarticular infections, which they will frequently encounter in their practice as orthopedic surgeons. Analyzing the teaching received by orthopedic residents in different multidisciplinary meetings and evaluating the teaching experiences of residents in other specialties during various multidisciplinary meetings would be interesting areas for further exploration.

## Conclusions

This study, although straightforward and having certain limitations, demonstrates that MTBMs, which are characterized by highly specialized discussions, also serve as a place of teaching in domains of learning that rely on the residents' roles and motivations. Depending on the specialties and desired learning objectives, it is worth considering how MTBM can be utilized to provide well-structured teaching experiences with clearly defined intended learning outcomes. Assessing the significance and value of MTBM in medical education could be achieved through an innovative prospective study in pedagogy.

## Appendices

**Table 1:** Study questionnaire

Question	Response Options
1. In which semester did you do the orthopedic oncology rotation?	[Text Response]
2. If you had to choose 3 items that would best qualify the MTBM for you:	1. distressing 2. Challenging 3. Difficult 4. Routine 5. Useless for my training 6. Useful for my training 7. Interesting 8. Exciting 9. Place of teaching 10. Good working atmosphere
3.Do you think MTBM is an appropriate place for teaching?	Totally disagree =1 Disagree = 2 Don't know =3 Agree =4 Totally agree = 5
4. How did MTBM benefit you the most (rank from most to least)	1- Learning how to present a case 2- Building confidence in public speaking 3- Knowledge of the pathology treated by MTBM (sarcoma or bone metastasis management) 4- Knowledge of radiologic imagery reading 5- General knowledge in medical oncology and/or radiotherapy and/or pathology.
5. Did MTBM allow you to get to know the different specialists involved in MTBM better and give you a better knowledge of the hospital establishment?	Totally disagree =1 Disagree = 2 Don't know =3 Agree =4 Totally agree = 5
6. Has MTBM helped you acquire useful skills or knowledge applicable to your daily life (regardless of the field)?	Totally disagree =1 Disagree = 2 Don't know =3 Agree =4 Totally agree = 5
7. Do you believe that having participated in MTBM will provide you with easier access to tumor board for case presentations if needed in your future practice?	Totally disagree =1 Disagree = 2 Don't know =3 Agree =4 Totally agree = 5
8. As part of the training for orthopedic residents, would you recommend that your colleagues undertake a 3-month rotation in orthopedic oncology?	Totally disagree =1 Disagree = 2 Don't know =3 Agree =4 Totally agree = 5
9. Do you think we can improve the teaching during the MTBM?	Totally disagree =1 Disagree = 2 Don't know =3 Agree =4 Totally agree = 5
10. Do you have any ideas in a few words to improve teaching during MTBM?	[Text Response]
